# Restorative Effect of Microalgae *Nannochloropsis oceanica* Lipid Extract on Phospholipid Metabolism in Keratinocytes Exposed to UVB Radiation

**DOI:** 10.3390/ijms241814323

**Published:** 2023-09-20

**Authors:** Michał Biernacki, Tiago Conde, Anna Stasiewicz, Arkadiusz Surażyński, Maria Rosário Domingues, Pedro Domingues, Elżbieta Skrzydlewska

**Affiliations:** 1 Department of Analytical Chemistry, Medical University of Bialystok, Kilinskiego 1, 15-069 Bialystok, Poland; michal.biernacki@umb.edu.pl (M.B.); anna.stasiewicz@umb.edu.pl (A.S.); 2 Mass Spectrometry Centre, LAQV-REQUIMTE, Department of Chemistry, University of Aveiro, Santiago University Campus, 3810-193 Aveiro, Portugal; tiagoalexandreconde@ua.pt (T.C.); mrd@ua.pt (M.R.D.); p.domingues@ua.pt (P.D.); 3 CESAM—Centre for Environmental and Marine Studies, Department of Chemistry, University of Aveiro, Santiago University Campus, 3810-193 Aveiro, Portugal; 4 Department of Medicinal Chemistry, Medical University of Bialystok, Kilinskiego 1, 15-069 Bialystok, Poland; arkadiusz.surazynski@umb.edu.pl

**Keywords:** microalgae *Nannochloropsis oceanica*, keratinocytes, fatty acids, lipid peroxidation, endocannabinoids, eicosanoids

## Abstract

Ultraviolet B (UVB) radiation induces oxidative stress in skin cells, generating reactive oxygen species (ROS) and perturbing enzyme-mediated metabolism. This disruption is evidenced with elevated concentrations of metabolites that play important roles in the modulation of redox homeostasis and inflammatory responses. Thus, this research sought to determine the impacts of the lipid extract derived from the *Nannochloropsis oceanica* microalgae on phospholipid metabolic processes in keratinocytes subjected to UVB exposure. UVB-irradiated keratinocytes were treated with the microalgae extract. Subsequently, analyses were performed on cell lysates to ascertain the levels of phospholipid/free fatty acids (GC-FID), lipid peroxidation byproducts (GC-MS), and endocannabinoids/eicosanoids (LC-MS), as well as to measure the enzymatic activities linked with phospholipid metabolism, receptor expression, and total antioxidant status (spectrophotometric methods). The extract from *N. oceanica* microalgae, by diminishing the activities of enzymes involved in the synthesis of endocannabinoids and eicosanoids (PLA2/COX1/2/LOX), augmented the concentrations of anti-inflammatory and antioxidant polyunsaturated fatty acids (PUFAs), namely DHA and EPA. These concentrations are typically diminished due to UVB irradiation. As a consequence, there was a marked reduction in the levels of pro-inflammatory arachidonic acid (AA) and associated pro-inflammatory eicosanoids and endocannabinoids, as well as the expression of CB1/TRPV1 receptors. The microalgal extract also mitigated the increase in lipid peroxidation byproducts, specifically MDA in non-irradiated samples and 10-F4t-NeuroP in both control and post-UVB exposure. These findings indicate that the lipid extract derived from *N. oceanica*, by mitigating the deleterious impacts of UVB radiation on keratinocyte phospholipids, assumed a pivotal role in reinstating intracellular metabolic equilibrium.

## 1. Introduction

One of the alternatives to the pharmacological treatment of dermatological conditions using synthetic compounds is the utilization of preparations of a natural origin. These natural compounds are typically distinguished by their significantly reduced adverse effects in comparison to synthetic pharmaceuticals [1]. Natural agents have found application in protecting against the deleterious effects of solar radiation, particularly UVB radiation, which leads to erythema, cellular damage, melanin accumulation, and a substantial increase in both epidermal and dermal thickness [2]. Furthermore, UVB radiation disrupts cellular metabolism, primarily in epidermal cells, including keratinocytes [3,4]. Prior investigations have elucidated that the primary consequence of UVB radiation exposure is the increased generation of reactive oxygen species (ROS), which, through modifications to the structure of biologically active macromolecules, notably proteins, typically result in alterations to their biological activity [4,5]. Consequently, the activity of antioxidant proteins may be diminished [6]. In response to this scenario, there is an increase in the activity of the transcription factor Nrf2, which is responsible for orchestrating the biosynthesis of antioxidant and cytoprotective proteins [7]. Since both Nrf2 and Nrf2-related proteins interact with the pro-inflammatory transcription factor NF-κB, oxidative stress typically coincides with the activation of inflammatory processes resulting from increased NF-κB transcription factor activity. This disruption in cellular metabolism exacerbates various inflammatory signals that ultimately lead to apoptosis [4,8]. Given the daily exposure of human skin to solar radiation and its detrimental effects, continuous efforts are made to identify protective and/or regenerative agents, particularly those with less potential for adverse effects. These agents include substances that can act simultaneously in multiple mechanisms, making them suitable for use in products designed to protect the epidermis and its cells from the harmful consequences of UVB radiation. Among the many natural sources of biologically active compounds, algae, encompassing both microalgae and macroalgae, is progressively garnering attention [9,10]. For instance, the microalgae *Nannochloropsis oceanica* has been identified as a source of numerous compounds with both anti-inflammatory and antioxidant properties. These compounds include proteins, amino acids, lipids, carbohydrates, and vitamins (A, B1, B2, B12, C, D, E, and K), as well as carotenoids (β-carotene, lutein, violaxanthin, zeaxanthin, and astaxanthin) [11,12]. Microalgal proteins also contain several antioxidant enzymes, such as superoxide dismutase, catalase, peroxidase, and glutathione reductase, which can participate in antioxidant defense mechanisms [13]. Microalgae also contains non-enzymatic compounds with antioxidant attributes. Bioactive constituents of *Nannochloropsis oceanica* have been demonstrated to modulate skin inflammation processes, including those in skin fibroblasts, by directly influencing the transcription factor NF-κB and MAP kinases, as well as indirectly by reducing the activity of lipolytic enzymes, such as the inducible COX isoform (COX-2) and nitric oxide synthase (NOS). Consequently, these bioactive compounds modify cellular lipid mediator levels [14,15]. Additionally, *Nannochloropsis oceanica* contains lipids that serve as carriers of omega-3 fatty acids, predominantly EPA, constituting approximately 3% of the algal biomass [16]. These bioactive lipids may play a pivotal role in regulating lipid mediator levels in the dermis and red blood cells of humans following EPA supplementation [17,18]. Consequently, both algal biomass and extracts are increasingly being employed in the treatment of dermatological conditions, for example, the use of macroalgae in thalassotherapy [19] and the incorporation of algae rich in essential omega-6 and omega-3 PUFAs in skin care products [20].

Drawing from previous literature [9,21], it has been suggested that marine algae, particularly *Nannochloropsis oceanica*, and more specifically, its lipid extracts, may modulate the metabolism of polyunsaturated fatty acids (PUFAs), whether free or esterified to phospholipids. This modulation results in alterations in the levels of lipid mediators of the eicosanoid and endocannabinoid groups, which may contribute to the regulation of both redox balance and the reduction in inflammation, both of which are disrupted in skin cells exposed to UVB radiation.

Consequently, the objective of this study was to assess the impact of a lipid extract derived from the microalgae *Nannochloropsis oceanica* on the metabolism of PUFAs, whether free or esterified to phospholipids, of the keratinocyte membranes. These alterations are influenced by ROS and lipolytic enzymes, which may ultimately provide insight into new therapeutic strategies for human skin constant exposure to solar radiation.

## 2. Results

### 2.1. Impact of the Lipid Extract Derived from the Microalgae Nannochloropsis oceanica on the Antioxidant Status of Keratinocytes

**Figure 1:**
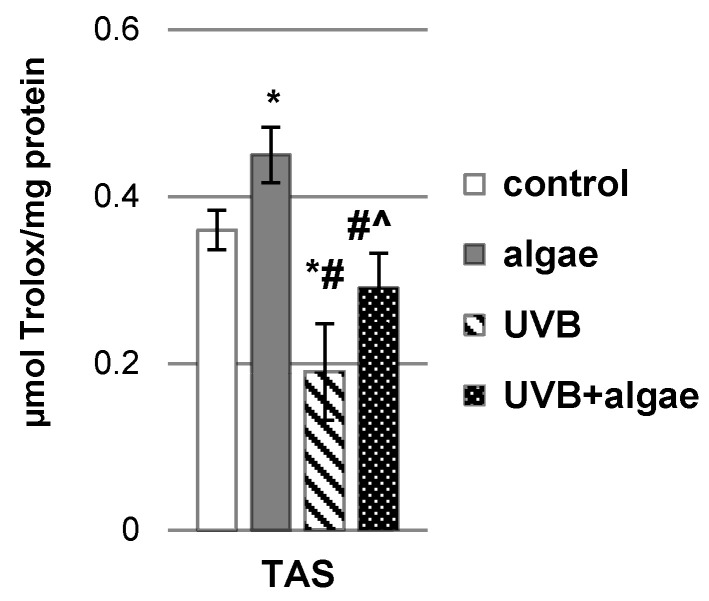
The influence of *Nannochloropsis oceanica* microalgae extract on the total antioxidant status (TAS) in various keratinocyte groups: control (n = 5); cultured with algae extract for 24 h (n = 5); irradiated with UVB (60 mJ/cm^2^) (n = 5); and irradiated with UVB (60 mJ/cm^2^) and subsequently cultured for 24 h with algae extract (n = 5). Mean values ± standard deviation (SD) and statistically significant differences for *p* < 0.05 are depicted: *—vs. control group; #—vs. algae group; and ^—vs. UVB group.

The total antioxidant status (TAS) of keratinocytes following exposure to UVB radiation was assessed, revealing a significant reduction in cellular antioxidant capacity by approximately 50% (Figure 1). Interestingly, when keratinocytes were incubated with the lipid extract from *Nannochloropsis oceanica*, there was a notable enhancement in TAS. Specifically, in both the control group with algae supplementation and following UVB radiation exposure with extract treatment, TAS exhibited an increase of approximately 25% and 53%, respectively, in comparison to the control and UVB-exposed groups.

### 2.2. Impact of Nannochloropsis oceanica Microalgae Lipid Extract on Keratinocyte Lipid Metabolism

**Figure 2:**
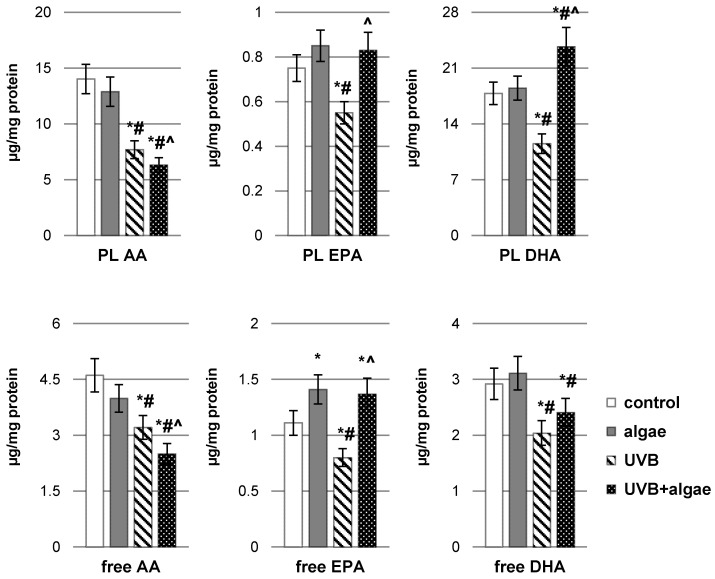
The impact of *Nannochloropsis oceanica* microalgae extract on the levels of unsaturated phospholipid fatty acids and free fatty acids, including arachidonic acid (AA), eicosapentaenoic acid (EPA), and docosahexaenoic acid (DHA), in various keratinocyte groups: control (n = 5); cultured with algae extract for 24 h (n = 5); irradiated with UVB (60 mJ/cm^2^) (n = 5); and irradiated with UVB (60 mJ/cm^2^) and subsequently cultured with algae extract for 24 h (n = 5). Mean values ± standard deviation (SD) and statistically significant differences for *p* < 0.05 are denoted as follows: *—vs. control group; #—vs. algae group; and ^—vs. UVB group.

Upon UVB irradiation of keratinocytes, a statistically significant reduction in the levels of both free and esterified polyunsaturated fatty acids (PUFAs), including arachidonic acid (AA), docosahexaenoic acid (DHA), and eicosapentaenoic acid (EPA), was observed (Figure 2). Conversely, in non-UVB-irradiated keratinocytes, incubation with the lipid extract derived from *Nannochloropsis oceanica* microalgae resulted in an elevation of free EPA levels, while leaving the levels of other PUFAs unchanged. However, in the presence of oxidative stress induced with UVB radiation, cells treated with the algae extract exhibited increased levels of both free and esterified PUFAs, specifically DHA and EPA. Conversely, the utilization of the microalgae extract was linked to a decrease in the concentration of arachidonic acid (AA), a precursor of eicosanoids.

**Figure 3:**
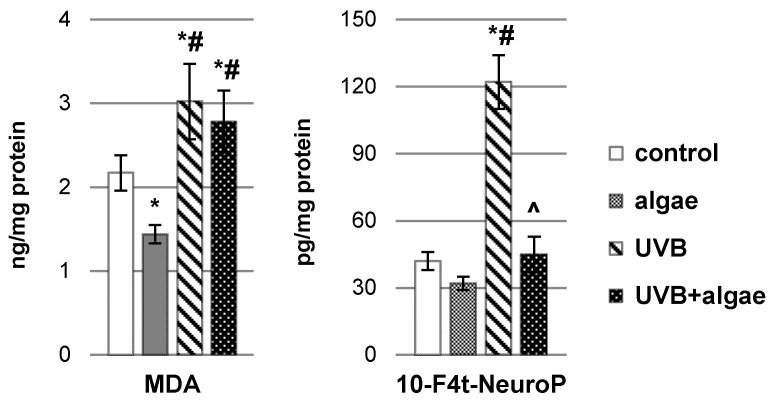
The impact of *Nannochloropsis oceanica* microalgae extract on the levels of lipid peroxidation products, including malondialdehyde (MDA) and 10(RS)-10-F4t-neuroprostane (10-F4t-NeuroP), in various keratinocyte groups: control (n = 5); cultured with algae extract for 24 h (n = 5); irradiated with UVB (60 mJ/cm^2^) (n = 5); and irradiated with UVB (60 mJ/cm^2^) and subsequently cultured with algae extract for 24 h (n = 5). Mean values ± standard deviation (SD) and statistically significant differences for *p* < 0.05 are indicated as follows: *—vs. control group; #—vs. algae group; and ^—vs. UVB group.

The decrease in total antioxidant status (TAS) within keratinocytes under the influence of UVB radiation occurred with an increased ROS-dependent metabolism of polyunsaturated fatty acids (PUFAs). This phenomenon was corroborated with the elevation in the levels of lipid peroxidation products, notably malondialdehyde (MDA) resulting from the process of different PUFAs’ peroxidation as well as neuroprostanes, 10(RS)-10-F4t-neuroprostane (10-F4t-NeuroP), generated mainly as a result of the peroxidation of phospholipid docosahexaenoic acid (Figure 3). Intriguingly, the administration of a lipid extract derived from *Nannochloropsis oceanica* microalgae not only mitigated the levels of MDA and neuroprostanes in control keratinocytes but also, as a result of effective regeneration of cells irradiated with UVB, ultimately reduced their levels, especially in the case of neuroprostanes.

**Figure 4:**
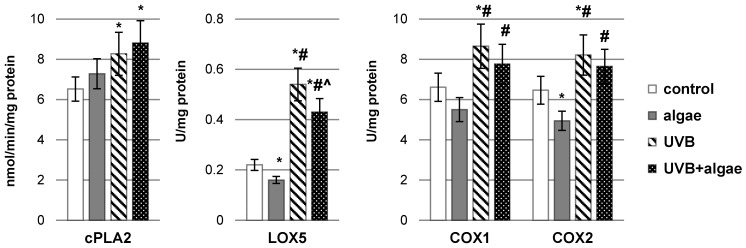
The impact of *Nannochloropsis oceanica* microalgae extract on the activity of cytosolic phospholipase A2 (cPLA2), lipoxygenase-5 (LOX-5), and cyclooxygenase-1/2 (COX-1/2) in various keratinocyte groups: control (n = 5); cultured with algae extract for 24 h (n = 5); irradiated with UVB (60 mJ/cm^2^) (n = 5); and irradiated with UVB (60 mJ/cm^2^) and subsequently cultured with algae extract for 24 h (n = 5). Mean values ± standard deviation (SD) and statistically significant differences for *p* < 0.05 are represented as follows: *—vs. control group; #—vs. algae group; and ^—vs. UVB group.

**Figure 5:**
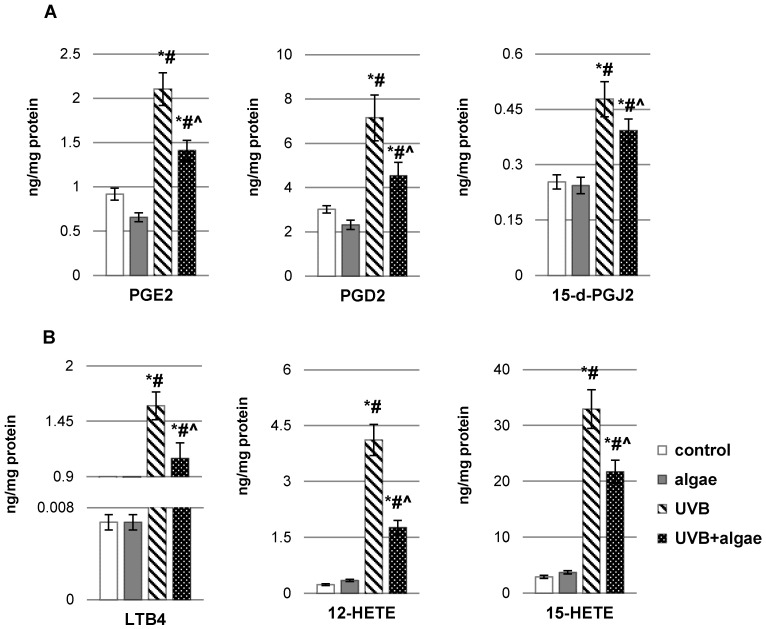
The influence of *Nannochloropsis oceanica* microalgae extract on the levels of (**A**) eicosanoids produced by cyclooxygenases (COXs) (COX1/2), including prostaglandin E2 (PGE2), prostaglandin D2 (PGD2), and 15-deoxy-delta12,14-prostaglandin J2 (15d-PGJ2), and (**B**) eicosanoids produced by lipoxygenases (LOXs), encompassing leukotriene B4 (LTB4), 12-hydroxyeicosatetraenoic acid (12-HETE), and 15-hydroxyeicosatetraenoic acid (15-HETE), in various keratinocyte groups: control (n = 5); cultured with algae extract for 24 h (n = 5); irradiated with UVB (60 mJ/cm^2^) (n = 5); and irradiated with UVB (60 mJ/cm^2^) and subsequently cultured with algae extract for 24 h (n = 5). Mean values ± standard deviation (SD) and statistically significant differences for *p* < 0.05 are indicated as follows: *—vs. control group; #—vs. algae group; and ^—vs. UVB group.

The lipid extract from microalgae *Nannochloropsis oceanica* had a notable influence on the enzymatic metabolism of polyunsaturated fatty acids (PUFAs), notably through the reduction in inducible cyclooxygenase isoform (COX-2) activity and lipoxygenase-5 (LOX-5) activity in control keratinocytes as well as LOX-5 activity in UVB-irradiated keratinocytes (Figure 4). Nonetheless, UVB radiation perturbed the metabolism of phospholipids and free PUFAs in keratinocytes, causing alterations in the activity of enzymes involved in phospholipid and free PUFA metabolism (PLA2, COX1/2, and LOX-5). This resulted in an increase in the activity of enzymes responsible for the biosynthesis of eicosanoids (COXs and LOX-5) in response to UVB radiation, consequently leading to an increased generation of these lipid mediators (Figure 5). The levels of all eicosanoids in UVB-irradiated keratinocytes catalyzed by COX (PGE2, PGD2, and 15d-PGJ2), and those of LTB4, 12-HETE, and 15-HETE catalyzed by LOX, exhibited elevation following UVB irradiation. While the algae extract did not significantly impact the level of eicosanoids in non-irradiated cells, it did partially mitigate the increased generation of all previously mentioned eicosanoids under pathophysiological conditions.

**Figure 6:**
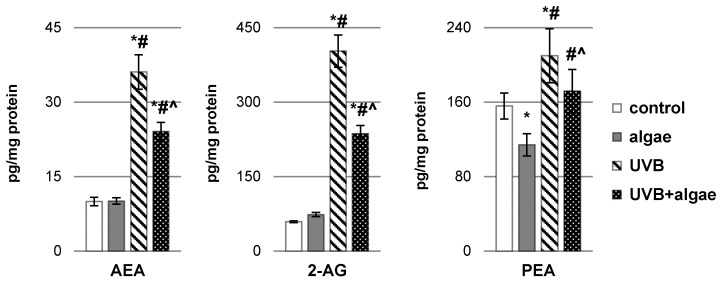
The impact of *Nannochloropsis oceanica* microalgae extract on the levels of endocannabinoids, including anandamide (AEA), 2-arachidonoylglycerol (2-AG), and the related compound palmitoylethanolamide (PEA), in various keratinocyte groups: control (n = 5); cultured with algae extract for 24 h (n = 5); irradiated with UVB (60 mJ/cm^2^) (n = 5); and irradiated with UVB (60 mJ/cm^2^) and subsequently cultured with algae extract for 24 h (n = 5). Mean values ± standard deviation (SD) and statistically significant differences for *p* < 0.05 are indicated as follows: *—vs. control group; #—vs. algae group; and ^—vs. UVB group.

In this investigation, we also examined the levels of other lipid mediators, specifically endocannabinoids and their derivatives (Figure 6), known for their capacity to modulate both oxidative stress and inflammatory signals. After exposure to UVB radiation, there was a notable elevation in the levels of classic endocannabinoids, N-arachidonoylethanolamine (AEA) and 2-arachidonoylglycerol (2-AG), as well as a derivative of palmitoylethanolamide (PEA), within keratinocytes. Conversely, in control cells treated with the microalgae extract, no significant alterations in the levels of AEA and 2-AG were detected, except for a reduction in the level of PEA. However, in keratinocytes subjected to UVB radiation and subsequently treated with microalgae *Nannochloropsis oceanica* extracts, a significant reduction in the levels of the tested endocannabinoids and the PEA derivative was observed.

**Figure 7:**
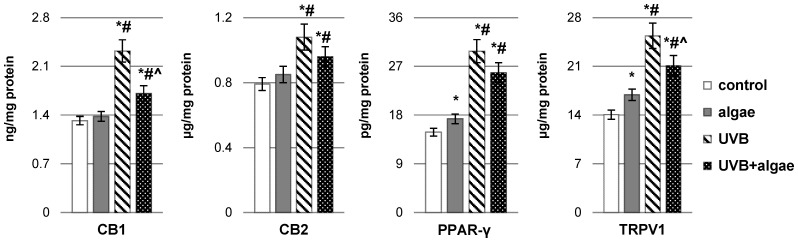
The impact of *Nannochloropsis oceanica* microalgae extract on the levels of receptors, including cannabinoid receptor type 1 (CB1), cannabinoid receptor type 2 (CB2), peroxisome proliferator-activated receptor gamma (PPAR-γ), and transient receptor potential cation channel subfamily V member 1 (TRPV1), in various keratinocyte groups: control (n = 5); cultured with algae extract for 24 h (n = 5); irradiated with UVB (60 mJ/cm^2^) (n = 5); and irradiated with UVB (60 mJ/cm^2^) and subsequently cultured with algae extract for 24 h (n = 5). Mean values ± standard deviation (SD) and statistically significant differences for *p* < 0.05 are indicated as follows: *—vs. control group; #—vs. algae group; and ^—vs. UVB group.

Under the influence of UVB radiation, the expression of CB1 and CB2 cannabinoid receptors and PPAR-γ and TRPV1 receptors increases, which can be agonized by both endocannabinoids and eicosanoids (Figure 7). The introduction of the microalgae extract into the keratinocyte culture medium resulted in an upregulation of the expression of PPAR-γ and TRPV1 receptors. Conversely, a tendency toward reduced expression of all tested receptors was observed in cells exposed to UVB radiation and subsequently treated with the algae extract. Nevertheless, a significant reduction was noted only for CB1 and TRPV1 receptors.

## 3. Discussion

Exposure to UV radiation causes both short- and long-term metabolic effects in the skin, which lead to erythema and photoaging, but also increase the risk of skin cancer [22]. The physicochemical barrier against UV rays, especially UVB, is primarily established by epidermal cells, including keratinocytes, Merkel cells, and melanocytes, all of which play roles in the skin’s immune mechanisms [23,24,25,26,27]. Merkel cells produce serotonin, met-enkephalin, and the vasoactive intestinal peptide, contributing to the skin’s neuroendocrine system and homeostasis maintenance. Additionally, Merkel cells secrete the CD200 protein, which holds significance in the inflammatory process [25]. In contrast, melanocytes exhibit an immunomodulatory effect, achieved through melanin biosynthesis that reduces the production of pro-inflammatory cytokines while interacting with T lymphocytes and fibroblasts. Furthermore, melanocytes respond to external factors by releasing immunomodulators like inducible nitric oxide synthase and pro-inflammatory cytokines, influencing nearby cells such as keratinocytes and fibroblasts and activating lymphocytes [26].

It is well-established that UVB radiation triggers increased reactive oxygen species (ROS) production in skin cells. When combined with impaired antioxidant mechanisms, this results in cellular redox imbalance, leading to structural and functional modifications in essential cellular components, including proteins and membrane phospholipids [28]. Consequently, this situation promotes the release of signaling molecules that induce keratinocyte proliferation as well as trigger a pro-inflammatory response and/or ultimately leads to cell death [3,28,29,30,31]. The findings of this study align with this phenomenon, as they demonstrate a reduction in keratinocyte antioxidant capacity (assessed as TAS) under the influence of UVB radiation, subsequently altering the metabolism of membrane phospholipids, influenced by both ROS and lipolytic enzymes (PLA2, COX1/2, and LOX-5). It is important to note that the activity of these enzymes increases in conditions of oxidative stress, as previously reported [32,33]. This shift contributes to elevated levels of lipid mediators, including lipid peroxidation products (MDA and 10-F4t-NeuroP), as well as enzymatic reaction products, such as eicosanoids and endocannabinoids, consistent with prior research [32,34].

The increase in endocannabinoid levels, which act as agonists for G-protein-coupled membrane receptors, particularly cannabinoid receptors (CB1 and CB2), results in the modulation of redox balance and inflammatory signaling within cells [35]. Furthermore, AEA, one of the endogenous TRPV1 agonists, along with ROS and lipid peroxidation products (which are elevated with UVB radiation), regulate TRPV1 expression by oxidizing its thiol groups. This underscores the close relationship between TRPV1 receptor activity and oxidative stress, consequently affecting inflammatory signaling [36,37]. Conversely, the PPARγ receptor plays a role in modulating inflammatory signaling by inducing the p65 subunit of the NF-κB transcription factor [38]. Consequently, the obtained results validate previous reports of increased oxidative stress and inflammatory responses in UVB-exposed keratinocytes [28,30,31].

To alleviate the unfavorable metabolic changes resulting from exposing keratinocytes to UVB, these cells after irradiation were treated with the microalgae Nannochloropsis oceanica lipid extract. This marine alga is a rich source of various bioactive compounds, including antioxidants, polysaccharides, triglycerides, lipids, and vitamins, which makes it a promising candidate for the regeneration of metabolic processes caused by, among others, daily exposure to solar radiation [11,12,39]. The microalgae *N. oceanica* employed in this study contains compounds with both antioxidant and anti-inflammatory properties, including proteins, amino acids, lipids, carbohydrates, vitamins, and carotenoids [11,12,39]. Additionally, the sonication of algae before introduction into a cell promotes the formation of liposomes constituting vesicles with lipid bilayers with a hydrophobic interior and hydroxyl heads on the outside, which allows for the transfer of compounds with hydrophobic and hydrophilic properties, respectively [40,41,42]. They are an effective means of delivering physicochemically differentiated components of microalgae extracts into cells, including compounds with antioxidant and anti-inflammatory properties [40]. It is worth noting that liposomes can traverse the membranes of human skin cells, such as keratinocytes, and release the components of algae, including proteins, polyunsaturated fatty acids (PUFAs), hormones, and vitamins, into the intracellular milieu [43,44]. The outcomes of this study validate that liposomes from the *N. oceanica* extract increase the antioxidant capacity of keratinocytes both under control conditions (cells + microalgae) and after UVB irradiation (cells + UVB + microalgae), which may confirm the supporting role of microalgae antioxidants in the metabolic activities of control keratinocytes, and also indicate the effective regenerative and antioxidant effects of microalgae components on keratinocytes exposed to UVB radiation. This confirms the significant presence and effectiveness of antioxidant compounds in the liposomes from the *N. oceanica* extract.

The comprehensive lipid composition of the extract used in this study has been previously documented [21]. This extract contains an array of lipids, including glycolipids such as mono- and diacylglycerol, 3-O-40-(N,N,N-trimethyl)homoserine, sulfoquinosyldiacylglycerol, and mono- and digalactosyldiacylglycerol. Additionally, it comprises phospholipids, notably phosphatidylcholine, phosphatidylethanolamine, phosphatidylglycerol, and phosphatidylinositol, alongside neutral lipids. Moreover, due to its high sphingolipid and ceramide concentration, this extract readily interacts with skin cells, which also contain these constituents within their intercellular matrix [45,46].

Microalgae further contains carotenoids, including the lipophilic astaxanthin, recognized for its potent antioxidant properties. Through the activation of Nrf2, astaxanthin elevates the expression of classical cellular antioxidant enzymes [47,48]. Similarly, omega-3 polyunsaturated fatty acids (ω-3 PUFAs), integral components of microalgae lipids, exert similar effects [49]. Furthermore, previous reports suggest that irrespective of the specific ingredients found in algae, such as the ethanolic extract of the macroalgae *Carpomitra costata*, they have the capacity to enhance the antioxidant levels in keratinocytes exposed to UVB radiation [50].

Previous research has demonstrated that algae extracts, such as ethyl acetate extracts from the microalgae *Ettlia* sp. YC001, contain antioxidant compounds, being able to diminish ROS levels, thus shielding human dermal fibroblasts from the repercussions of UVB radiation [50,51]. These prior findings indicate that the augmented ROS levels induced with UVB radiation [3] facilitate the increased production of non-enzymatic oxidative modifications of PUFAs in keratinocytes. These modifications involve low-molecular-weight, electrophilic aldehydes (4-HNE and MDA) and products arising from the oxidative cyclization of PUFAs, such as F2-isoprostanes and neuroprostanes [3,28]. The results obtained in our study are consistent with previous reports, revealing an elevated level of MDA and neuroprostanes as a consequence of UVB radiation.

Phospholipids are the primary target of UVB-induced oxidation, with the potential to affect the biological properties of cell membranes, including a reduction in their integrity and consequently the onset of metabolic disruptions within cells [52]. As a consequence, the regeneration of phospholipids by the microalgae may be important for maintaining the physiological functions of the skin. Microalgae N. oceanica contains lipids that serve as carriers of omega-3 fatty acids, primarily EPA, which possesses robust antioxidant and anti-inflammatory properties [53,54]. This may partially explain the observed decrease in pro-oxidant activity in control, non-irradiated cells after application of the lipid extract. Moreover, keratinocytes exposed to UVB radiation and then exposed to microalgae show a reduction in the level of phospholipid peroxidation products. However, considering that proteins and partly DNA can also undergo structural and functional changes under the influence of UVB radiation [31], the regenerative effect of algae can also be indicated to proteins, which was also observed in previous studies [34].

Oxidative stress induced with UVB radiation also augments the activity of enzymes responsible for metabolizing phospholipids and PUFAs (PLA2, COX1/2, and LOX-5) into lipid mediators, including endocannabinoids and eicosanoids [32]. The interconnectedness of these bioactive mediators implies that their actions within cells can be viewed as part of a broader metabolic network governing inflammatory signals, inflammation, and redox balance at the level of individual keratinocytes in a cell culture, but potentially also playing a role in regulating metabolic equilibrium throughout the entire skin [32,55].

In this study, we demonstrated that the levels of all tested eicosanoids, including those generated under the influence of COXs (PGE2, PGD2, and 15-d-PGJ2) and LOX (LTB4, 12-HETE, and 15-HETE), encompassing both anti-inflammatory (15-d-PGJ2 and 15-HETE) and pro-inflammatory (PGE2, PGD2, LTB4, and 12-HETE) compounds, were elevated in keratinocytes exposed to UVB radiation, in accordance with previous research [33,55,56]. On the other hand, the application of the *N. oceanica* lipid extract after UVB irradiation reduced the levels of prostaglandins PGE2, PGD2, and 15-d-PGJ2. This reduction may be related to the tendency to decrease the activity of enzymes involved in their biosynthesis (COX-1/2). Existing literature data suggest that ethanolic and aqueous extracts from the microalgae *P. tricornutum* inhibited the activity of COX-2 in lipopolysaccharide-stimulated human peripheral blood mononuclear cells, underscoring their anti-inflammatory properties [57]. Furthermore, our previous studies have indicated that a lipid extract from the microalgae *N. oceanica* in keratinocyte cells reduced the level of NF-κB [34], a regulator of COX-2 expression [58]. Additionally, the observed reduction in LOX-5 activity under the influence of the microalgae extract contributes to the decrease in eicosanoids such as LTB4 and 12- and 15-HETE acids’ level. Literature data suggest that several compounds found in microalgae may be implicated in modulating the levels of eicosanoids, including EPA, which constitutes 3% of the biomass of *N. oceanica* [16]. Its elevated level in keratinocytes, particularly following treatment with UVB and the microalgae extract, implies that EPA competes with AA as a substrate in enzymatic metabolism catalyzed by COX and LOX enzymes, potentially reducing the generation of AA metabolites in keratinocytes [17,53].

Another class of compounds discovered in microalgae that exhibit the ability to modulate eicosanoid biosynthesis comprise fat-soluble vitamins. Notably, vitamin E has demonstrated the capacity to attenuate the enzymatic activity of LOX-5 and COX isoenzymes, consequently contributing to the downregulation of LTB4, PGD2, and PGE2 levels, as corroborated with in vitro investigations (utilizing RAW264.7 macrophages, lung epithelial cells, and isolated platelets) [59,60,61]. Similar observations have been made in the context of inhibiting the synthesis of pro-inflammatory eicosanoids, such as LTB4 and TBX2, in avian blood [62]. Conversely, the oral supplementation of B vitamins in humans promotes the elevation of polyunsaturated fatty acids (PUFAs), including linoleic acid (LA), α-linolenic acid (ALA), arachidonic acid (AA), eicosapentaenoic acid (EPA), and docosahexaenoic acid (DHA), thereby exerting an influence on eicosanoid levels [63].

Another set of compounds encountered in *N. oceanica* extracts comprises carotenoids, including β-carotene, lutein, and zeaxanthin, which possess the capacity to enhance the anti-inflammatory activity of keratinocytes through integration into the lipid bilayer of cellular membranes. This integration increases the skin’s defenses against the deleterious consequences of UV radiation [64,65]. Furthermore, lutein has the capability to diminish levels of reactive oxygen species (ROS) while impeding the ROS-dependent activation of NF-κB and STAT3. This dual action leads to a reduction in the generation of inflammatory mediators, encompassing IL-1β, IL-6, MCP-1, TNF-α, COX2, and iNOS, consequently reducing inflammatory processes [66]. In addition, lutein, particularly in conjunction with EPA and DHA, can downregulate the activity of lipolytic enzymes, resulting in diminished levels of eicosanoids such as PGE2, LTB4, and LTC4 [67]. A similar metabolic response is plausible in the case of keratinocytes treated with *Nannochloropsis oceanica* lipids.

It has been described that the increased presence of prostaglandin PGE2 in UVB-irradiated keratinocytes was substantially mitigated using the application of a microalgae extract [68]. The overproduction of PGE2 induced with UV radiation can elicit immunosuppression, thereby fostering increased cellular proliferation and inflammatory signaling, ultimately potentiating the likelihood of neoplastic transformation [69]. Additionally, PGE2 inhibits collagen production and induces the expression of matrix metalloproteinase 1 (MMP1) through the augmentation of ERK1/2 phosphorylation, consequently contributing to accelerated skin ageing [70]. Conversely, prostaglandin PGD2 and its metabolite 5-deoxy-12,14-prostaglandin J2 (15d-PGJ2) assume a pivotal role in the regulation of inflammatory signals [71]. 15d-PGJ2 has the capability to modulate signaling pathways involving transcription factors NF-κB and Nrf2, the functionality of which is subject to alteration by the lipid extract derived from the microalgae *N. oceanica* [34,71,72,73]. The increased levels of both compounds following UVB irradiation of keratinocytes were significantly attenuated following treatment with the *N. oceanica* extract. The *N. oceanica* extract additionally reduced the activity of LOX-5 induced with UVB radiation, consequently leading to a reduction in the levels of lipid mediators, including LTB4, 12-HETE, and 15-HETE [32], the latter of which serves as a precursor to anti-inflammatory lipoxins [74]. Elevated levels of LTB4 stimulate the expression of the BLT2 receptor, precipitating augmented ROS generation and apoptosis [75]. Consequently, the diminishment of LOX-5 activity, a pivotal enzyme in LTB4 production, may accelerate skin healing [76].

Endocannabinoids, specifically anandamide (AEA), 2-arachidonoylglycerol (2-AG), and their derivative palmitoylethanolamide (PEA), constitute another category of lipid mediators with a regulatory role in oxidative stress and inflammatory signals. In UVB-irradiated keratinocytes, there was an observed elevation in the levels of these endocannabinoids. However, the application of the *N. oceanica* extract effectively mitigated the heightened levels of all the aforementioned endocannabinoids. This effect may be attributed to the notable presence of eicosapentaenoic acid (EPA) within the extract, which could potentially inhibit endocannabinoid biosynthesis by downregulating mRNA expression of N-acyl phosphatidylethanolamine-specific phospholipase D (NAPE-PLD), the key enzyme involved in endocannabinoid biosynthesis [18,77].

The UVB-induced upregulation of endocannabinoids and eicosanoids in keratinocytes augments the impact of these lipid mediators on cellular metabolism, including redox equilibrium and inflammatory signaling. This is achieved through the enhanced expression of G-protein-coupled receptors, encompassing cannabinoid receptors (CB1 and CB2), transient receptor potential cation channel subfamily V member 1 (TRPV1), and peroxisome proliferator-activated receptor-gamma (PPARγ) [36]. Endocannabinoids like AEA and 2-AG act as agonists for CB1 and CB2 receptors, as well as ligands for TRPV1 and PPAR-γ receptors, mirroring the behavior of PEA [35]. All endocannabinoids contribute to the modulation of oxidative stress and its repercussions, including lipid peroxidation, through mechanisms such as free radical scavenging and enhanced expression of CB1 and CB2 receptors [36]. Notably, CB1 receptor enhanced expression has been associated with increased reactive oxygen species (ROS) production and tumor necrosis factor-alpha (TNF-α) release, while CB2 receptor enhanced expression exerts the opposite effects [36].

The significant reduction in CB1 receptor expression observed following the application of the *N. oceanica* extract suggests a potential reduction in both oxidative stress and inflammatory signaling in keratinocytes. This finding is consistent with previous results demonstrating, a decrease in TNF-α levels [34]. However, it is noteworthy that both the endocannabinoids and eicosanoids assessed in this study, as well as the constituents of *N. oceanica*, including EPA and lutein, also serve as agonists for TRPV1 and PPAR-γ receptors [78]. This potentially supports the increased expression of these receptors in control cells treated with the microalgae extract, contributing to their anti-inflammatory and antioxidant attributes. Oxylipin 15-deoxy-delta12,14-prostaglandin J2 (15d-PGJ2) serves as a natural agonist for PPARγ receptors, consequently exerting antioxidant, anti-inflammatory, antifibrotic, and antiangiogenic effects [71]. Additionally, agonists for TRPV1 and PPARγ receptors include 12-HETE and 15-HETE acids, and LTB4 [79,80]. Hence, the augmented expression of TRPV1 and PPARγ receptors may be attributed to the cumulative impact of all lipid mediators from *N. oceanica* on these receptors. Conversely, the introduction of a microalgae lipid extract to UVB-irradiated keratinocyte cultures resulted in a reduction in the expression of receptors that enhance pro-oxidant and pro-inflammatory effects, namely CB1 and TRPV1.

## 4. Materials and Methods

### 4.1. Materials and Cell Culture Treatment

#### 4.1.1. Preparation of Microalgae Lipid Extracts

For the extraction of lipids from *Nannochloropsis oceanica* algae, a modified Folch method [21,81] was employed. Lipids were extracted from 25 mg of a spray-dried *N. oceanica* biomass, which was acquired from an aquaculture in autotrophic conditions (provided by Allmicroalgae Natural Products S.A., situated in Pataias, Portugal). The biomass, displaying a green coloration, was utilized as the starting material.

The extraction process involved the addition of a dichloromethane/methanol solvent mixture (2:1, *v*/*v*) to the algae biomass. Subsequently, the resulting mixture was subjected to vortexing and centrifugation (at 670× *g* for 10 min). The supernatant was carefully separated, and this sequence of operations was repeated two additional times. The combined supernatants underwent drying under a stream of nitrogen, followed by reconstitution in a solution comprising dichloromethane and methanol. After vortexing, Milli-Q water was introduced into the mixture. Upon another centrifugation step (at 670× *g* for 10 min), the organic phase was isolated and collected. Further extractions of the aqueous phase were carried out twice. The lipid extract utilized in the study was generated by combining the organic phases.

The lipid content was quantified gravimetrically, and the lipid profile of the resulting *Nannochloropsis oceanica* algae extract was assessed using hydrophilic interaction liquid chromatography combined with high-resolution mass spectrometry (HILIC-MS) and tandem MS (MS/MS). This analysis employed an Orbitrap Q-Exactive hybrid quadrupole mass spectrometer (Thermo Fisher Scientific, Waltham, MA, USA), as previously detailed in an earlier publication [21]. A comprehensive summary of the composition of the *N. oceanica* lipid extract is provided in Supplementary Table S1.

#### 4.1.2. Cell Culture

CDD 1102 KERTr (CRL2310) human immortalized keratinocytes obtained from the American Type Culture Collection ATCC^®^ (Manassas, VA, USA) were used for the study. Cells from passage 9 were grown in a humidified atmosphere of 5% CO_2_ and 37 °C. Keratinocyte-growth-medium–SFM supplemented with a 1% bovine pituitary extract (BPE) and antibiotics, 50 U/mL of penicillin and 50 μg/mL of streptomycin, was used. All experiments were performed under completely sterile conditions, using sterile plastics and cell culture reagents purchased from Gibco (Grand Island, NY, USA).

The keratinocytes, after reaching 70% confluency, were exposed to UVB radiation. The radiation dose was 60 mJ/cm^2^ (312 nm, power density at 4.08 mW/cm^2^) (Bio-Link Crosslinker BLX 312/365, Vilber Lourmat, Germany), which corresponded to approximately 70% of cell survival as measured with the MTT (3-(4,5-dimethylthiazol-2-yl)-2,5-diphenyltetrazolium bromide) method [82] and, as previously shown, leads to the activation of pro-oxidative conditions [28]. To avoid heat stress, cells were irradiated in cold PBS (phosphate-buffered saline, 4 °C) and the distance of the plates from lamps was constantly maintained at 15 cm. After irradiation, keratinocytes were washed with a medium and were treated with the lipid extract from *N. oceanica* algae in 0.1% DMSO (dimethyl sulfoxide) at concentrations of 3 μg/mL, which did not induce changes in cell viability compared to control cells measured with the MTT test [82]. Control cells were incubated in parallel for 24 h with algae extracts under standard conditions (without irradiation) in a medium containing the lipid extracts from *N. oceanica* in 0.1% DMSO. After 24 h of incubation, all cells were washed with PBS, harvested by scraping in cold PBS, and, after disintegration, centrifuged; the resulting solution was used for biochemical assays. The total protein content of the cell lysate was measured using the Bradford assay [83].

The entire experiment was conducted with four distinct experimental groups:

I: control (n = 5)—keratinocytes (passage 9) cultured in a standard medium;

II: algae (n = 5)—keratinocytes (passage 9) cultured for 24 h in a medium containing the lipid extract from *N. oceanica* algae (3 μg/mL);

III: UVB (n = 5)—keratinocytes (passage 9) irradiated with UVB (60 mJ/cm^2^), and following exposure to UVB radiation, cells were incubated for 24 h in a standard medium;

IV: UVB + algae (n = 5)—keratinocytes (passage 9) irradiated with UVB (60 mJ/cm^2^), and following exposure to UVB radiation, cells were incubated for 24 h in a medium supplemented with the microalgae *N. oceanica* lipid extract (3 μg/mL).

### 4.2. Methods

#### 4.2.1. Estimation of Total Antioxidant Status (TAS)

Total antioxidant status (TAS) of keratinocytes was determined with a commercial kit using the 2,2-azinobis-(3-ethybenzodiazoline) 6-sulfonic acid (ABTS) radical cation assay according to the manufacturer’s protocol (Randox, Kearneysville, WV, USA). The ABTS test allows for determining the total effectiveness of lipophilic and hydrophilic antioxidants [84]. Pre-generated with oxidation of ABTS (2,29-azinobis-(3-ethyl-benzothiazoline- 6-sulfonic acid) by potassium persulfate, the colored radical monocation 2,29-azinobis-(3-ethylbenzothiazoline-6-sulfonic acid) (ABTS+•) was reduced in the presence of hydrogen-donating antioxidants. The reduction of ABTS+• reflects the antioxidant potential of the sample. For this purpose, the keratinocyte lysate was added to the ABTS+• working solution in a 96-well plate at 37 °C and the absorbance reduction was analyzed at 734 nm (Infinite 200; TECAN, Männedorf, Switzerland) for 10 min. The test results are reported with reference to the equivalent antioxidant capacity of Trolox (TEAC) as a standard. Trolox (Hoffman-LaRoche, Basel, Switzerland) is a water-soluble derivative of vitamin E with antioxidant properties and does not interact with other cellular components. The TAS level is therefore expressed in µmol of Trolox per mg of protein.

#### 4.2.2. Determination of Phospholipid and Free PUFA Levels

The phospholipid and free fatty acids were analyzed with gas chromatography [85]. Fatty acids were extracted with the Folch procedure using a chloroform/methanol mixture (2:1, *v*/*v*) with 0.01% butylated hydroxytoluene. Free fatty acids and total phospholipids were separated using TLC. The mobile phase was 100 mL of a 60:40:3 (*v*/*v*/*v*) heptane–diisopropyl ether–acetic acid solution. Fatty acids were converted to the corresponding methyl esters (FAMEs) with boron trifluoride in methanol. The FAMEs were analyzed with GC using a capillary column coated with the Varian CP-Sil88 stationary phase (50 m × 0.25 mm, ID of 0.2 μm, Varian). The column was fitted on a Clarus 500 Gas Chromatograph (Perkin Elmer, Waltham, MA, USA). The injector and flame ionization detector (FID) temperatures were set at 240 °C. Peak identification was accomplished by comparing peak retention times to those of two internal standards (nonadecanoic acid (19:0) and 1,2-dinonadecanoyl-sn-glycero-3-phosphocholine (19:0 PC)). The levels of PUFAs were determined using a calibration curve range of 1–100 µg/mL for AA (r^2^ − 0.9994); of 0.1–10 µg/mL for EPA (r^2^ − 0.9991); of 1–100 µg/mL for DHA (r^2^ − 0.9997); and converted for mg of protein. The levels of AA (LLQ = 0.5 µg/mg of protein), EPA (LLQ = 0.05 µg/mg of protein), and DHA (LLQ = 0.5 µg/mg of protein) were expressed in μg/mg of protein.

#### 4.2.3. Determination of the Level of Lipid Peroxidation Products

Lipid peroxidation in the cell culture was estimated by measuring small-molecular-weight reactive aldehyde–malondialdehyde (MDA) as well as neuroprostanes (10-F4t-NeuroP).

The MDA level was determined using gas chromatography coupled with mass spectrometry (7890A GC–7000 with a triple quadrupole mass spectrometer, Agilent Technologies, Palo Alto, CA, USA) as the O-pentafluorobenzyl-oxime (O-PFB-oxime) or O-pentafluorobenzyl-oxime-trimethyl silane (O-PFB-oxime-TMS) derivatives, based on a method of Tsikas and co-workers with minor modifications [86]. Hydroxynonenal-d3 (d3-4-HNE; 50 pmol) was added as an internal standard. MDA was separated using an HP-5 ms capillary column and analyzed in the selected ion monitoring mode (SIM). After incubation with methanol, samples were deproteinized by 24 h. Aldehyde was extracted with hexane. The hexane layer was evaporated and N,O-bis-(trimethylsilyl)-trifluoroacetamide in 1% trimethylchlorosilane was added. Then, 1 µL aliquots were loaded onto the column. The following ions were monitored: *m*/*z* 204.0 and 178.0 for MDA-PFB and *m*/*z* 245.0 for IS (d3-4-HNE) derivatives. The level of MDA was determined using a calibration curve range of 0.2–20 ng/mL (r^2^ − 0.9997) and converted for mg of protein. The level of MDA (LLQ = 50 pg/mg of protein) was expressed in ng/mg of protein.

Neuroprostanes’ (NPs) level (measured as 10-F4t-NeuroP) was quantified using the modified LC-MS method of Dupuy [87]. Quality and quantity determinations were carried out with ultra-performing-liquid-chromatography–tandem-mass-spectrometry with an electrospray ionization source (ESI) (LCMS 8060, Shimadzu, Kyoto, Japan). Analyte separation was performed using an Eclipse Plus C18 analytical column (2.1 × 100 mm; a 1.8 µm particle size) with a 3 µL injection volume. The mobile phase consisted of (A) 0.1% acetic acid in MilliQ water and (B) acetonitrile. The separation was performed using a linear gradient with water/acetic acid (99.5:0.5, *v*/*v*) and acetonitrile. Briefly, 10-F4t-NeuroP was isolated using the SPE method, after an alkaline hydrolysis step. 8-isoPGF2α–d4 was used as an internal standard. 10-F4t-NeuroP was analyzed in the negative ion mode using MRM. Transitions of the precursor to the production *m*/*z* 357.2→197.1 and 377.0→153.0 were used for 8-isoPGF2α-d4 and 10-F4t-NeuroP, respectively. The level of 10-F4t-NeuroP was determined using a calibration curve range of 5–300 pg/mL (r^2^ − 0.9991) and converted for mg of protein. The level of 10-F4t-NeuroP (LLQ = 1 pg/mg of protein) was expressed in pg/mg of protein.

#### 4.2.4. Determination of the Activity of Lipolytic Enzymes

The activity of enzymes involved in the metabolism of phospholipids and fatty acids was examined spectrophotometrically using commercially available assay kits, phospholipase A2 (PLA2–EC.3.1.1.4; Cayman Chemical Company, Ann Arbor, MI, USA), cyclooxygenases 1 and 2 (COX-1/2–EC.1.14.99.1; Cayman Chemical Company, Ann Arbor, MI, USA), and lipoxygenase-5 (LOX; Sigma-Aldrich, St. Louis, MO, USA), following the manufacturer’s instructions.

The samples were incubated with arachidonoyl thio-phosphatidylcholine, a synthetic substrate of cPLA2. The hydrolysis of arachidonoylthio-phosphatidylcholine by PLA2 released the free thiol, which was converted to NTB via a reaction with DTNB. The NTB concentration was determined with a spectrophotometric analysis at 405 nm [88]. PLA2 activity was expressed in nmol of arachidonoyl thio-phosphatidylcholine/min/mg of protein.

Cayman’s COX colorimetric inhibitor screening assay measures the peroxidase components of COXs. The peroxidase activity was assessed colorimetrically by monitoring the appearance of oxidized N,N,N′- and N′-tetramethyl-phenylenediamine (TMPD) at 590 nm [89]. One unit of COX’s enzyme activity is defined as the amount of enzyme that will oxidize 1.0 nmol of TMPD per minute at 25 °C. The specific COX1-inhibitor SC-560 was applied to measure only COX-2 activity. Cyclooxygenases’ activities were expressed in U/mg of protein.

In the Sigma-Aldrich lipoxygenase-5 (LOX-5) activity assay, lipoxygenase converts the LOX substrate to an intermediate that reacts with the probe generating a fluorescent product. The increase in the fluorescent signal can be recorded at Ex/Em 500/536 nm and is directly proportional to LOX-5 activity. One unit (U) of LOX-5 was determined as the amount of enzyme that causes oxidation of 1 µmol of the LOX probe per minute at pH 7.4 and at room temperature. LOX-5 activity was expressed in U/mg of protein.

#### 4.2.5. Determination of the Level of Endocannabinoids

An analysis of endocannabinoids (anandamide (AEA) and 2-arachidonylglycerol (2-AG)) and related compounds (palmitoylethanolamide (PEA)) was performed using a Shimadzu UPLC system (Nexera X2) coupled with an electrospray ionization source (ESI) to a Shimadzu 8060 Triple Quadrupole system (Shimadzu, Kyoto, Japan) operating in the positive ion mode [90]. Analyte separation was performed using a 120 EC-C18 analytical column (3.0 × 150 mm; a 2.7 µm particle size) with a 5 µL injection volume. The mobile phase consisted of (A) 0.1% formic acid in MilliQ water and (B) 0.1% formic acid in acetonitrile. The following gradient was employed: 0.0–5.0 min, 70–80% B; 5.0–10.0 min, 80–88% B; 10.0–16.0 min, 78–100% B; 16.0–20.0 min, 100% B; 20.0–21.0 min, 100–70% B; and 21.0–25.0 min, 70% B. Briefly, cell culture samples were thawed on ice and spiked with 10 µL of an internal standard solution (100 ng/mL of AEA-d8, 2-AG-d8, and OEA-d4) and then applied to pre-washed and conditioned solid phase extraction SPE cartridges. After loading the sample, the cartridges were washed, dried under a high vacuum, and eluted. Eluates were concentrated and reconstituted in 30 μL of ACN/H_2_O (7:3) with 0.1% formic acid and vortexed (if necessary, they were centrifuged to remove any residuals). Solutions were then transferred to LC vials with low-volume inserts and an LC–MS/MS analysis was performed immediately. Transitions of the precursors to the product ions were as follows: *m*/*z* 348.3→62.15 for AEA, *m*/*z* 379.3→287.25 for 2-AG, *m*/*z* 300.3→62.0 for PEA, *m*/*z* 356.2→63.05 for AEA-d8, *m*/*z* 387.3→294.0 for 2-AG-d8, and *m*/*z* 330.20→66.15 for OEA-d4. The level of endocannabinoids was determined using a calibration curve range of 1–100 pg/mL for AEA (r^2^ − 0.9992); of 10–1000 pg/mL for 2-AG (r^2^ − 0.9995); of 10–500 pg/mL for PEA (r^2^ − 0.9993); and converted for mg of protein. The levels of AEA (LLQ = 1 pg/mg of protein), 2-AG (LLQ = 2 pg/mg of protein), and PEA (LLQ = 0.2 pg/mg of protein) were expressed in pg/mg of protein.

#### 4.2.6. Determination of the Level of Eicosanoids

An eicosanoid analysis was performed using a Shimadzu UPLC system (Nexera X2) coupled with an electrospray ionization source (ESI) to a Shimadzu 8060 Triple Quadrupole system (Shimadzu, Kyoto, Japan) operating in the negative mode [91]. Analyte separation was performed using an Eclipse Plus C18 analytical column (2.1 × 100 mm; a 1.8 µm particle size) with a 3 µL injection volume. The mobile phase consisted of (A) 0.1% acetic acid in MilliQ water and (B) acetonitrile. The following gradient was employed: 0.0–1.0 min, 25–40% B; 1.0–2.5 min, 40–42% B; 2.5–4.5 min, 42–50% B; 4.5–10.5 min, 50–65% B; 10.5–12.5 min, 65–75% B; 12.5–14.0 min, 75–85% B; 14.0–14.5 min, 85–95% B; 14.5–15 min, 95–25% B; and 15.0–16.0 min, 25% B. Briefly, samples were thawed on ice and spiked with a 10 µL internal standard solution (100 ng/mL of LTB4-d4, PGD2-d4, 15-d-PGJ2-d4, and 15-HETE-d8) and then applied to pre-washed and conditioned solid phase extraction SPE cartridges. After loading the sample, the cartridges were washed, dried under a high vacuum, and eluted. Eluates were concentrated and reconstituted in 30 μL of ACN/H_2_O (8:2) with 0.1% acetic acid and vortexed (if necessary, they were centrifuged to remove any residuals). Solutions were then transferred to LC vials with low-volume inserts and an LC–MS/MS analysis was performed immediately. The precursor to the product-ion transition was as follows: *m*/*z* 351.3→271.2 for PGE2 and PGD2, 315.2→271.2 for 15-d-PGJ2, *m*/*z* 335.2→195.1 for LTB4, *m*/*z* 319.2→179.1 for 12-HETE, *m*/*z* 319.0→301.2 for 15-HETE, *m*/*z* 355.0→275.3 for PGD2-d4, *m*/*z* 319.3→275.2 for 15-d-PGJ2-d4, *m*/*z* 339.1→197.1 for LTB4-d4, and 327.0→226.2 for 15-HETE-d8. The level of eicosanoids was determined using a calibration curve range of 0.1–10 ng/mL for PGE_2_ (r^2^ − 0.9996); of 0.5–25 ng/mL for PGD_2_ (r^2^ − 0.9991); of 0.05–5 ng/mL for 15d-PGJ_2_ (r^2^ − 0.9992); of 0.001–10 ng/mL for LTB_4_ (r^2^ − 0.9998); of 0.05–20 ng/mL for 12-HETE (r^2^ − 0.9999); of 0.5–100 ng/mL for 15-HETE (r^2^ − 0.9995); and converted for mg of protein. The levels of PGE_2_ (LLQ = 2 pg/mg of protein), PGD_2_ (LLQ = 1 pg/mg of protein), 15d-PGJ_2_ (LLQ = 0.5 pg/mg of protein), LTB_4_ (LLQ = 5 pg/mg of protein), 12-HETE (LLQ = 1 pg/mg of protein), and 15-HETE (LLQ = 1 pg/mg of protein) were expressed in ng/mg of protein.

#### 4.2.7. Determination of Expression of Membrane Receptors

Protein expression membrane receptors were measured using an enzyme-linked immunosorbent assay (ELISA) (Nunc Immuno MaxiSorp, Thermo Fisher Scientific, Waltham, MA, USA) [92]. Plates with attached protein cell lysates were incubated with a blocking solution (5% fat-free dry milk in a carbonate binding buffer) at 4 °C for 3 h. Next, the samples were washed with PBS with 0.1% Tween 20, and incubated overnight with an appropriate primary antibody against TRPV1 (host: mouse) (Sigma-Aldrich, St. Louis, MO, USA); CB1 and CB2 (host: mouse) (Santa Cruz Biotechnology, Dallas, TX, USA); and PPARγ (host: rabbit) (Invitrogen, Thermo Fisher Scientific, Waltham, MA, USA). After washing with PBS with 0.1% Tween 20, samples were incubated with a peroxidase blocking solution (3% hydrogen peroxide, 3% fat-free dry milk in PBS) for 30 min at room temperature. The goat anti-rabbit/mouse EnVision+ Dual Link/HRP solution (1:100) (Agilent Technologies, Santa Clara, CA, USA) was used as a secondary antibody. Secondary antibodies were removed after 1 h of incubation at room temperature. Then, samples were incubated for 40 min with a chromogen substrate solution (0.1 mg/mL of TMB, 0.012% H_2_O_2_). The reaction was stopped by adding 2 M of sulfuric acid and absorption was read within 10 min at 450 nm and automatically recalculated from standard curves for each protein: 0.1–10 ng/mL (r^2^ − 0.999) for CB_1_; 0.1–6 µg/mL (r^2^ − 0.999) for CB_2_; 1–150 pg/mL (r^2^ − 0.999) for PPARγ; 1–100 µg/mL (r^2^ − 0.999) for TRPV1, and converted for mg of protein. (TRPV1: Lifespan Biosciences, Seattle, WA, USA; PPARγ: Fine, Test Wuhan, Hubei, China; CB1: Abcam, Cambridge, UK; and CB2: Abnova, Taipei, Taiwan.) The receptors were expressed as ng/mg of protein for CB1 (LLQ = 0.5 μg/mg of protein), pg/mg of protein for PPAR-γ (LLQ =5 pg/mg of protein), and μg/mg of protein for CB2 (LLQ = 0.2 μg/mg of protein) and TRPV1 (LLQ =5 μg/mg of protein).

#### 4.2.8. Statistical Analysis

Data were analyzed with a one-way analysis of variance (ANOVA) followed by post-hoc Tukey testing using Statistica software (Statistica 13.3, Stat Soft Polska, Cracow, Poland) and expressed as a mean ± SD. Values of *p* ≤ 0.05 were considered significant, and only these results are discussed in detail.

## 5. Conclusions

In summary, these investigations demonstrated the capacity of the lipid extract derived from the microalgae *Nannochloropsis oceanica* to regenerate keratinocytes previously exposed to UVB radiation. This protective effect primarily arises from its ability to counteract the decline in cellular antioxidant capacity, thereby protecting membrane phospholipids. The microalgae extract significantly mitigated the increased metabolism of phospholipids, consequently leading to a reduction in the production of eicosanoids and endocannabinoids. These lipid mediators, both directly and through receptor expression, are implicated in the modulation of oxidative and inflammatory conditions. As a result, the outcome was a diminishment in oxidative stress and inflammation, often accompanying solar radiation exposure.

Considering the above findings, the lipid extract sourced from the microalgae *Nannochloropsis oceanica* holds promise for incorporation as a natural ingredient in cosmetic products aimed at regenerating the skin after daily exposure to solar radiation. Additionally, there is potential for its inclusion in medical products aimed at mitigating oxidative stress and inflammation, which represent risks in the development of various skin disorders, including cancer. However, these applications will only be possible after verifying the effect of the *Nannochloropsis oceanica* microalgae extract on other cells of the epidermis, including melanocytes, and the dermis, including fibroblasts, as well as the evaluation of the response of skin cancer cell lines. Ultimately, in vivo research involving the application of the algae extracts directly on the skin of laboratory animals and clinical trials will also be necessary.

An issue that will also have to be taken into account when introducing the algae extract into cosmetic/medical applications is the need to standardize algae extracts, which may also pose a serious challenge for producing companies.

## Data Availability

All data generated or analyzed during this study are included in this published article.

## Supplementary Materials

The supporting information can be downloaded at: https://www.mdpi.com/article/10.3390/ijms241814323/s1.
